# Potential Therapeutic Effects of the Neural Stem Cell-Targeting Antibody Nilo1 in Patient-Derived Glioblastoma Stem Cells

**DOI:** 10.3389/fonc.2020.01665

**Published:** 2020-08-14

**Authors:** Gorjana Rackov, Giorgia Iegiani, Daniel Uribe, Claudia Quezada, Cristóbal Belda-Iniesta, Carmen Escobedo-Lucea, Augusto Silva, Pere Puig, Víctor González-Rumayor, Ángel Ayuso-Sacido

**Affiliations:** ^1^IMDEA Nanoscience, Madrid, Spain; ^2^Fundación de Investigación HM Hospitales, Madrid, Spain; ^3^Istitute of Applied Molecular Medicine, Faculty of Medicine, San Pablo CEU University, Madrid, Spain; ^4^Molecular Pathology Laboratory, Institute of Biochemistry and Microbiology, Faculty of Sciences, Universidad Austral de Chile, Valdivia, Chile; ^5^Instituto de Salud Carlos III, Madrid, Spain; ^6^Division of Pharmaceutical Biosciences, Faculty of Pharmacy, University of Helsinki, Helsinki, Finland; ^7^Wyss Institute for Biologically Inspired Engineering at Harvard University, Boston, MA, United States; ^8^Market Access Department, Merck Sharp & Dohme, Madrid, Spain; ^9^Atrys Health, Barcelona, Spain; ^10^Brain Tumor Laboratory, Fundación Vithas, Hospitales Vithas, Madrid, Spain; ^11^Instituto de Investigaciones Biosanitarias, Faculty of Experimental Sciences, Universidad Francisco de Vitoria, Madrid, Spain; ^12^Formerly, Fundación de Investigación HM Hospitales, Institute of Applied Molecular Medicine, Faculty of Medicine, San Pablo CEU University, Madrid, Spain

**Keywords:** Nilo1, antibody, glioblastoma, glioma stem cells, neural stem cells, immunotherapy

## Abstract

Glioblastoma (GBM) is the most devastating and least treatable brain tumor with median survival <15 months and extremely high recurrence rates. Promising results of immune checkpoint blockade obtained from pre-clinical studies in mice did not translate to clinic, and new strategies are urgently needed, particularly those targeting GBM stem cells (GSCs) that are held responsible for drug resistance and tumor recurrence. Patient-derived GSC cultures are critical for finding effective brain tumor therapies. Here, we investigated the ability of the recently described monoclonal antibody Nilo1 to specifically recognize GSCs isolated from GBM surgical samples. We employed five patient-derived GSC cultures with different stemness marker expression and differentiation potential, able to recapitulate original tumors when xenotransplanted *in vivo*. To answer whether Nilo1 has any functional effects in patient-derived GSCs lines, we treated the cells with Nilo1 *in vitro* and analyzed cell proliferation, cell cycle, apoptosis, sphere formation, as well as the expression of stem *vs.* differentiation markers. All tested GSCs stained positively for Nilo1, and the ability of Nilo1 to recognize GSCs strongly relied on their stem-like phenotype. Our results showed that a subset of patient-derived GSCs were sensitive to Nilo1 treatment. In three GSC lines Nilo1 triggered differentiation accompanied by the induction of p21. Most strikingly, in one GSC line Nilo1 completely abrogated self-renewal and led to Bax-associated apoptosis. Our data suggest that Nilo1 targets a molecule functionally relevant for stemness maintenance and pinpoint Nilo1 as a novel antibody-based therapeutical strategy to be used either alone or in combination with cytotoxic drugs for GSC targeting. Further pre-clinical studies are needed to validate the effectiveness of GSC-specific Nilo1 targeting *in vivo*.

## Background

Glioblastoma (GBM, World Health Organization grade IV glioma) is the most aggressive and least treatable brain tumor. Current therapy for newly diagnosed GBM includes maximal surgical resection followed by concurrent radiation therapy with temozolomide (TMZ) and subsequent adjuvant TMZ therapy. Despite this standard of care treatment, median overall survival has only been extended to 14.6 months and 5-year survival rates are less than 10% (1). In addition, there is no effective treatment at the time of recurrence, which occurs in most of the patients. Bevacizumab – a humanized monoclonal antibody against VEGF (vascular endothelial growth factor) – was the most promising therapeutic agent for recurrent GBM. However, clinical trials have shown that, while it prolongs progression-free survival for 3 months, this does not translate to increased overall survival (2, 3). In fact, anti-VEGF therapy results in increased tumor invasiveness at the time of progression, which challenges surgical resection of recurrent GBM (4) and possibly even worsens the quality of life (3).

Significant progress in immuno-oncology has led to new treatments, such as immune checkpoint blockade, CAR (chimeric antigen receptor) T cell therapy, cytokine therapy, oncolytic viruses and dendritic cell and peptide vaccines. Currently, immune checkpoint blockade utilizing monoclonal antibodies against PD-1 (programmed cell death-1) or its ligand (PD-L1) is being extensively studied in GBM clinical trials (e.g., NCT02336165, NCT02617589, NCT02550249, NCT02017717); however, their efficacy so far has been very limited. Only a small subset of patients (8%) showed objective responses in a trial of anti-PD-1 in recurrent GBM (5), and these responses were transient due to acquired resistance mechanisms (6). New candidate immunotherapeutics are thus needed to be used in combination with immune checkpoint blockade and overcome GBM resistance mechanisms.

The model of cancer initiating cells proposes that tumor growth depends on a small population of undifferentiated cells, termed cancer stem cells (CSCs) because of their self-renewal ability and multilineage differentiation potential (7, 8). Due to their slow cell cycle and overexpression of efflux pumps, CSCs are held responsible for driving tumor progression and recurrence after treatment with irradiation and cytotoxic drugs. In fact, such treatment might lead to CSC enrichment after eliminating other cancer cells. Identifying CSCs and specifically targeting signaling pathways responsible for maintenance of their tumor-initiating and stem cell properties are thus of high clinical relevance. Nonetheless, specific targeting of glioblastoma stem-like cells (GSCs) is still challenging, given that truly specific GSC markers have not been described thus far. GSCs express markers associated with neural stem cells (NSCs), such as CD133, Nestin, CD44 and CD90. Substantial evidence suggests that GSCs originate from NSCs that undergo malignant transformation and migrate from subventricular zone (SVZ) to distant regions of the brain (9–11). In accordance with this hypothesis, GSCs share many features with NSCs of the SVZ, like high proliferative and migration potential, association with vasculature and reciprocal communication with perivascular niche (12). Interestingly, key signaling pathways responsible for NSC maintenance, proliferation, differentiation and migration, like EGFR, PDGFR, p53 or PTEN, are frequently altered in GBM. Novel therapeutic strategies directed to target not only GSCs, but also their putative cells of origin – NSCs of the SVZ – are thus worth considering for clinical implementation (12).

Nilo1 (neural identification lineage from olfactory bulb) is a monoclonal antibody generated after immunization of hamsters with olfactory-bulb-derived mouse neurospheres (13). Nilo1 specifically marks NSCs and early progenitors in the mouse brain (13), however, it is also able to recognize a homologous antigen in human neurospheres derived from GBM patients (14). Nilo1 treatment arrests mouse neurosphere proliferation (13), suggesting that it might recognize functionally relevant molecule involved in NSC stem cell maintenance. Nonetheless, whether Nilo1 affects human GSC functions remained unknown.

The aim of this study was to analyze the effects of Nilo1 treatment in patient-derived GSC cultures, which represent indispensable *in vitro* model for GBM basic studies and drug development (15, 16). We previously characterized these cells and showed that they express stem cell markers, grow as 3D neurospheres in serum-free conditions, and form tumors when xenotransplanted to immunodeficient mice brain, recapitulating the phenotype and gene expression of the original tumor (17). Our previous study revealed that Nilo1 indeed recognizes human GSCs (14), however, in the present work we observed that the effects of Nilo1 varied between GSC lines derived from different patients. Namely, one GSC line was completely resistant to Nilo1 treatment, while four other lines were sensitive. In three of those lines, Nilo1 led to slowing down the cell cycle and triggered differentiation, which was accompanied by the induction of cell cycle inhibitor p21. Most strikingly, in one GSC line Nilo1 completely abrogated self-renewal and led to apoptosis, associated with the induction of Bax. Overall, our data show that Nilo1 targets a functionally relevant molecule for GSC maintenance and suggest that patient-derived GSCs can be stratified according to their differential Nilo1 sensitivity. This establishes Nilo1 as a potential therapeutic agent to be used in combination with existing immunotherapy to improve GBM clinical outcome.

## Methods

### Isolation of GSCs, Cell Culture, and Differentiation

Glioblastoma stem-like cells were isolated from five freshly obtained GBM samples. All patients gave informed consent and the use of tumor samples was approved by Hospital La Fe (Spain) Ethics Committee. All patient-derived GSCs used in this study have been previously characterized and have generated tumors when xenotransplanted into nude mice [Ref. (17), and unpublished data]. GSCs cell expansion was carried out in serum-free DMEM/F-12 supplemented with N2, 300 ng/ml hydrocortisone, 2 μg/ml heparin, 30 ng/ml triiodothyronine, 10 ng/ml EGF and 20 ng/ml FGF-2. GSCs were routinely allowed to form spheres during 10 days in culture, dissociated using Accutase and then split 1:10. Medium was replaced every 3–5 days. For differentiation, the GSCs were allowed to form spheres during 6 days and then the medium was replaced with differentiation medium, containing the same basal media supplemented with 10% FBS and lacking EGF and FGF-2. All experiments were performed in mycoplasma-free conditions.

### Mesenchymal Stem Cell Culture

Human adipose tissue samples were obtained at private plastic surgery clinic (Clinica Dra. Isabel Moreno) from lipoaspiration procedures from 8 healthy patients under surgery by aesthetic reasons, aged between 18 and 35, following written informed consent and ethical research project approval by both Clinica Dra Isabel Moreno and Hospital General Foundation in Valencia ethical boards under the research project of Dr. Escobedo-Lucea. All the patients were previously screened for human immunodeficiency virus (HIV), hepatitis C and other infectious diseases. Cells were obtained following the protocol established from Planat-Benard (18), with a few modifications. Briefly, samples were digested in a solution of 1 mg/ml collagenase type I from Clostridium Histolyticum (Gibco, Grand Island, NY, United States) for 90 min at 37°C. The cells were then washed with 0.5% of HSA in Hank’s balanced salt solution (Gibco, Grand Island, NY, United States) and after discarding mature adipocytes, seeded in culture flasks with growth medium, Dulbecco’s modified Eagle’s medium (Invitrogen) supplemented with human or bovine serum mesenchymal stem cell qualified (Gibco, Grand Island, NY, United States), in a humidified atmosphere of 95% air and 5% CO2 at 37°C. The medium was replaced every 3 days. When primary culture became subconfluent, cells were detached using Tryple (Invitrogen) and subcultured in growth medium.

### Fluorescence Confocal Microscopy

Glioblastoma stem-like cell tumorspheres or dissociated single cells were plated on Matrigel-coated coverslips, fixed in 4% paraformaldehyde for 10 min and blocked with 10% BSA/0.05% Tween for 1 h at room temperature. Primary Nilo1 monoclonal antibody was generated by the fusion of hamster B cells and the mouse myeloma X63Ag8 (13) and purified in CNB-CSIC (Madrid, Spain). Cells were incubated with Nilo1 1:100 overnight at 4°C, followed by 1:200 FITC-conjugated anti-hamster secondary antibody from BD. F-actin was stained with Phalloidin-iFluor 647 (1:40, 1 h at room temperature, Abcam) and nuclei with DAPI (1:5000, 10 min at room temperature, Sigma). For stem-like and differentiation markers we used anti-GFAP (1:500, Dako) and anti-OLIG2 (1:500, Millipore), followed by Alexa fluor 555 goat anti-rabbit antibody (1:1000) from Invitrogen. To estimate cell viability within the tumorspheres, 5 μg/ml 7-AAD (BioLegend) was used. Coverslips were mounted with Fluorsave (Calbiochem). The images were taken using the glycerol ACS APO 20x NA0.60 immersion objective of a confocal fluorescence microscope (SPE, Leica-Microsystems) and analyzed using FIJI software.

### Nilo1 Treatment and Cell Viability Assay

Glioblastoma stem-like cell tumorspheres or dissociated single cells were plated in 96-well plate at a density of 3000 cells per well and treated with Nilo1 monoclonal antibody or *InVivo*MAb hamster anti-mouse CD3ε (BioXCell) as irrelevant control, at a concentration of 0.5 mg/ml for indicated time points. For 7-day treatment, the media was replenished once on Day 3. Cell viability was assessed using CellTiter 96 AQ_*ueous*_ One Solution Cell Proliferation Assay from Promega, according to manufacturer’s instructions.

### Neurosphere Formation Assay

After tumorsphere dissociation, GSCs were plated in 96-well plate at a density of 50 cells per well and treated as above, with 6 replicates per condition. Neurospheres were counted and photographed after 21 days.

### RNA Extraction and RT-PCR Analysis

Total RNA was extracted with TRIzol (Sigma) according to the manufacturer’s instructions and 1 μg RNA was retro-transcribed using cDNA kit from Applied Biosciences. Real-time PCR was performed using TB Green Premix Ex Taq RR420 (Takara) and detected by ABI PRISM 7900HT (Applied Biosystems). Data were analyzed using SDS 2.4 software (Applied Biosystems), normalized to the expression of β-actin and represented as the fold-change with respect to controls. All primers were synthesized, desalted and purified by Sigma, and the sequences were as follows: *NES*, 5′-GAGGTGGCCACGTACAGG-3′ (forward) and 5′-AAGCTGAGGGAAGTCTTGGA-3′ (reverse); *PROM1*, 5′-GGAAACTAAGAAGTATGGGAGAACA-3′ (for- ward) and 5′-CGATGCCACTTTCTCACTGAT-3′ (reverse); *OLIG2*, 5′-AGCTCCTCAAATCGCATCC-3′ (forward) and 5′-ATAGTCGTCGCAGCTTTCG-3′ (reverse); *PDGFRA*, 5′-CC ACCTGAGTGAGATTGTGG-3′ (forward) and 5′-TCTTCAGG AAGTCCAGGTGAA-3′ (reverse); *S100B*, 5′-GGAAGGGGTG AGACAAGGA-3′ (forward) and 5′-GGTGGAAAACGTCGAT GAG-3′ (reverse); *GFAP*, 5′-GTGGTGAAGACCGTGGAGAT-3′ (forward) and 5′-GTCCTGCCTCACATCACATC-3′ (reverse); *MAP-2*, 5′-CCTGTGTTAAGCGGAAAACC-3′ (forward) and 5′-AGAGACTTTGTCCTTTGCCTGT-3′ (reverse).

### Flow Cytometry

Glioblastoma stem-like cell tumorspheres were dissociated using Accutase (5 min, 37°C) and cells were stained with anti-PDGFRA-PE (BD), -CD133-APC (Miltenyi Biotec), -CD24-FITC (BD) and -CD90-FITC (Beckman Coulter) at the dilution of 1:100. The cells were analyzed on Attune Acoustic Focusing Cytometer from Applied Biosystems. The data were analyzed using FlowJo software (Tristar).

### Apoptosis Analysis

Following Nilo1- or irrelevant control-treatment, the tumorspheres were dissociated with Accutase (5 min, 37°C), stained with Pacific Blue Annexin V Apoptosis detection kit (BioLegend) according to the manufacturer’s protocol, and analyzed by flow cytometry.

### Cell Cycle Analysis

To analyze cell cycle, dissociated tumorspheres were washed with PBS, permeabilized with detergent, stained with PI for 30 min at 37°C according to the manufacturer’s instructions (DNA-Prep Reagent Kit, Beckman Coulter) and analyzed by flow cytometry.

### Statistical Analysis

Statistical significance was determined by unpaired two-tailed Student’s *t*-test for comparisons between two groups, or by 1- or 2-way ANOVA for multiple comparisons, followed by Bonferroni *post hoc* test. Differences were considered significant when *p* < 0.05. All statistical analyses were conducted using Prism 8 software (GraphPad).

## Results

### Human GBM Neurospheres Stain Positively for Nilo1

**FIGURE 1:**
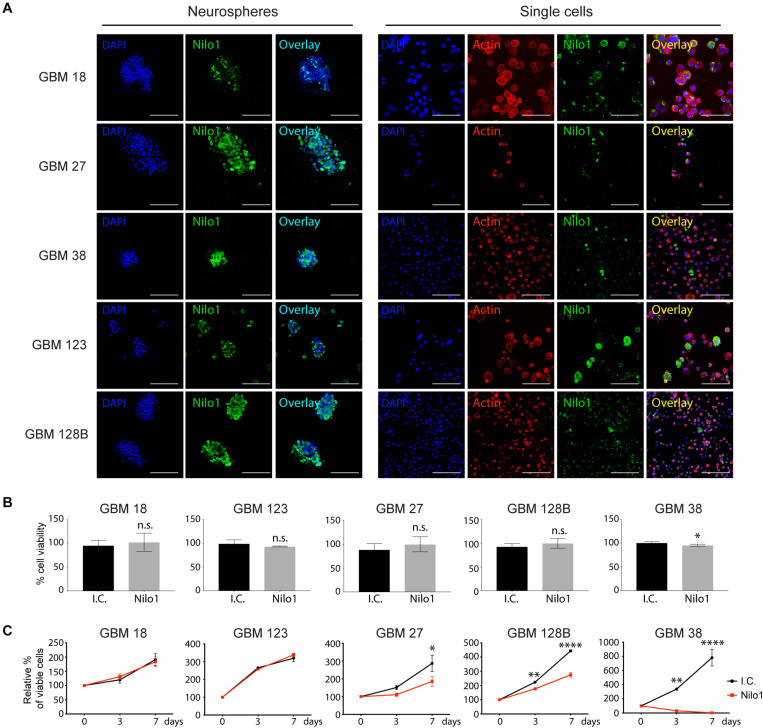
Nilo1 treatment reduces the viability of GSCs. **(A)** Confocal microscopy showing Nilo1-positive staining in five lines of patient-derived GSCs grown as neurospheres (left) or after dissociation to single cells (right). DAPI is shown in blue. Scale bar shows 100 μm. **(B)** MTS assay showing the effect of Nilo1 (0.5 mg/ml) 7-day-treatment on GSC tumorsphere viability compared with the i.c. (irrelevant control, hamster anti-mouse CD3ε). Data were normalized to i.c. treatment and show mean ± SD (*n* = 3), *p < 0.05, Student’s *t*-test. **(C)** GSCs were dissociated to single cells and treated with Nilo1 or i.c. (0.5 mg/ml) for 3 or 7 days. MTS assay showed a reduction in GSC viability after Nilo1 treatment. Data were normalized to day 0 and show mean ± SD (*n* = 3), *p < 0.05, **p < 0.01, ****p < 0.0001, two-way ANOVA (with Bonferroni correction).

Nilo1 has been shown to recognize NSCs in mice, as well as human GBM cells derived from patients. While Nilo1 arrests proliferation in mouse NSCs, whether Nilo1 has such an effect on human GSCs remained unknown. In this study, we used GSC-enriched cultures isolated from five GBM patients (GBM18, GBM27, GBM38, GBM123, and GBM128B) and grew them as neurospheres in growth-factor-enriched stem cell medium (19). Nilo1 gave positive staining in all five tested lines (Figure 1A, left panels), confirming our previous findings that Nilo1 recognizes human GBM neurospheres (14). To visualize cell morphology, neurospheres were dissociated into single cells and F-actin (filamentous actin) was stained with phalloidin. Although the relative abundance of Nilo1-positive cells varied between different GSC lines, only a small proportion of cells were positive for Nilo1 within each line (Figure 1A, right panels).

### Nilo1 Treatment Reduces Cell Viability and Self-Renewal Properties in a Subset of GSCs

To investigate the effect of Nilo1 stimulation on patient-derived GSCs, we allowed neurospheres to form during 10 days in stem cell culture, and then treated them for 7 days with 0.5 mg/ml Nilo1, the highest concentration that efficiently inhibited proliferation in mouse neurospheres (13). The numbers of viable cells were similar in Nilo1- and irrelevant antibody control-treated cells, except in GBM38, where Nilo1 treatment slightly reduced the numbers of viable cells compared with control (Figure 1B). Cell cycle analysis showed that during neurosphere formation G1 phase lengthens overtime and that the division cycles become longer resulting in decreased proportions of actively proliferating cells at Day 10 compared with Day 3 of cell culture (Supplementary Figure 1). We thus hypothesized that Nilo1 treatment might have an effect on the growth of single cells during neurosphere formation. To investigate this, we dissociated the neurospheres and treated them with Nilo1 or an irrelevant control antibody for 3 or 7 days. As before, Nilo1 didn’t show any effect on the viability of GBM18 and GBM123 cells compared with control (Figure 1C). However, in this setting Nilo1 treatment significantly reduced viable cell numbers in GBM27 and GBM128B cells (Figure 1C). Most strikingly, Nilo1 completely abrogated cell growth in GBM38 (Figure 1C). To exclude the possibility that Nilo1 treatment would be toxic to normal stem cells, we performed a 7-day-Nilo1 treatment on adipose tissue-derived mesenchymal stem cells. Our results confirmed that the viability of normal stem cells was not altered, suggesting that Nilo1 effects are restricted to GSCs (Supplementary Figure 2).

**FIGURE 2:**
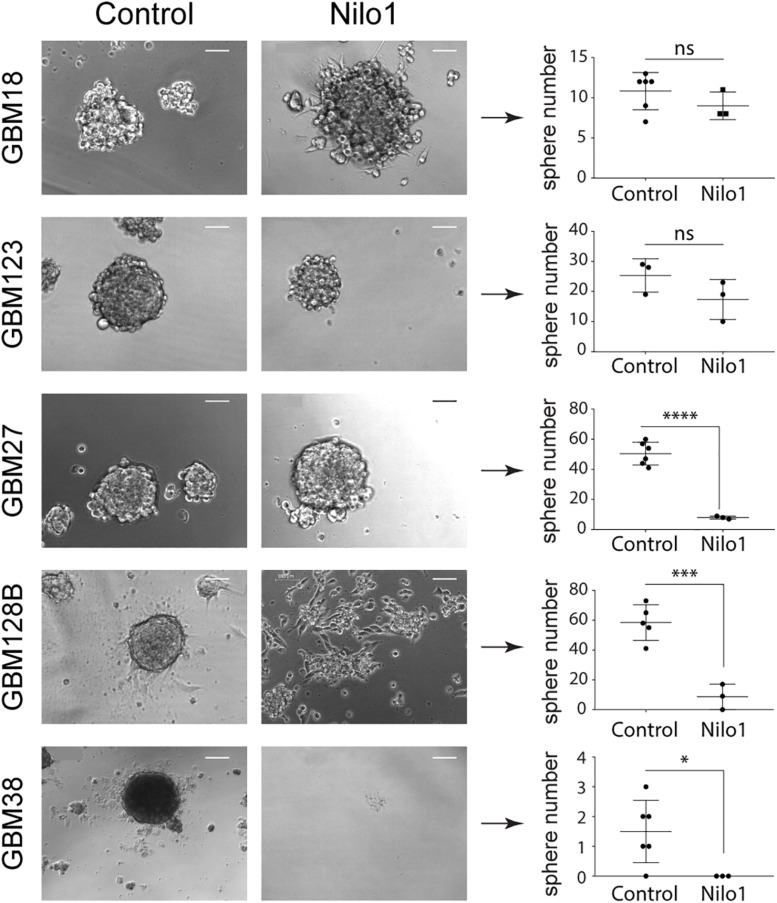
Nilo1 treatment reduces GSC self-renewal properties. For sphere-formation assay, GSC neurospheres were dissociated and 50 cells were plated per well in a 96-well plate. GSCs were treated with Nilo1 or irrelevant control (i.c.) at 0.5 mg/ml and spheres were counted after 3 weeks. Scale bar shows 100μm. Data show mean ± SD (*n* = 6), *p < 0.05, ***p < 0.001, ****p < 0.0001, Student’s *t*-test. NS, not significant.

Since Nilo1 treatment affected the viability of replicating single cells and not the fully formed spheres, we considered that Nilo1 might interfere with GSC self-renewal properties. We thus performed sphere-formation assay over the course of 3 weeks to investigate whether Nilo1 treatment would affect the ability of GSCs to form neurospheres. As in viability assay, Nilo1 didn’t show any effect on sphere formation in GBM18 and GBM123 cells compared with control treatment (Figure 2 and Supplementary Figure 3). However, Nilo1 effectively reduced GSC stemness in GBM27, GBM38 and GBM128B, as indicated by decreased number of spheres at 21 days after plating (Figure 2 and Supplementary Figure 3). Increased proportions of adherent cells were observed in Nilo1-treated GBM18 and GBM128B (Figure 2 and Supplementary Figure 3), suggesting that Nilo1 might interfere with sphere formation by inducing cell differentiation. Again, most striking effect was detected in GBM38, where not a single sphere could be observed after the Nilo1 treatment (Figure 2). To discard the possibility that some GSC lines were particularly sensitive to Nilo1 treatment due to suboptimal growing conditions and/or cell death, we performed 7-AAD staining of GBM27, GBM38, and GBM128B control neurospheres to check for the presence of non-viable cells at the outermost neurosphere layers as a sign of non-optimal growth (20). Nonetheless, we didn’t detect a significant number of 7-AAD-positive cells in external layers of these neurospheres, suggesting they were healthy and viable (Supplementary Figure 4). Collectively, our data showed that Nilo1 stimulation significantly reduces stemness in three out of five tested patient-derived GSC lines.

### Differentiation Reduces the Ability of Nilo1 to Recognize GSCs

**FIGURE 3:**
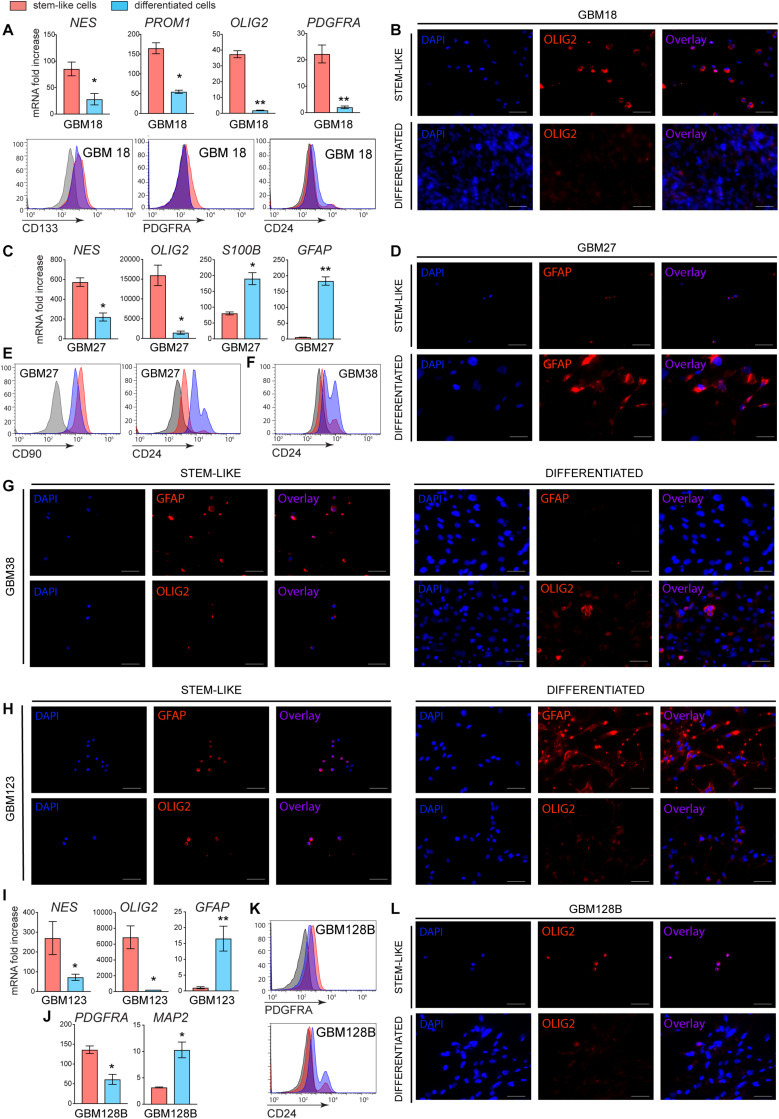
Stemness and differentiation markers in stem-like and differentiated GSCs. GSCs were grown in FBS-free media supplemented with growth factors for 10 days (stem-like cells). To induce differentiation, GSCs were grown in the same media for 6 days to form tumorspheres and then changed to growth-factor-free media supplemented with 10% FBS for additional 4 days (differentiated cells). **(A)** RT-PCR and flow cytometry analyses of mRNA and surface protein expression for GBM18. **(B)** Immunofluorescence analysis showing OLIG2 levels in GBM18. **(C)** RT-PCR analysis and **(D)** immunofluorescence showing the induction of astrocyte markers in GBM27. Flow cytometry showing increased CD24 surface levels in GBM27 **(E)** and GBM38 **(F)** after differentiation. **(G)** Immunofluorescence showing a decrease in GFAP and an increase in OLIG2 levels in GBM38 after differentiation. **(H)** Immunofluorescence analysis of GBM123, showing an increase in GFAP and decrease in OLIG2 levels after differentiation. **(I)** RT-PCR showing increased GFAP expression in GBM123. **(J)** RT-PCR and **(K)** flow cytometry showing decreased PDGFRA and increased neuronal markers MAP2 and CD24 in GBM128B following differentiation. **(L)** Immunofluorescence showing a decrease in OLIG2 levels in differentiated GBM128B cells. Gene expression analysis was done for all GSC lines at once and the data were normalized to GBM38, so the relative fold change can be appreciated between different GSC lines. Data show mean ± SD (*n* = 3), **p < 0.01, ***p < 0.001, ****p < 0.0001, Student’s *t*-test. For flow cytometry, negative staining control is in gray. Shown are representative histogram plots of two experiments performed. For immunofluorescence, white bar shows 50 μm. Shown are representative images of two independent experiments performed.

Our data showed that Nilo1 affects GSC self-renewal properties, which suggested that Nilo1 specifically targets cells in stem-like state. To investigate this, we next asked whether Nilo1 capacity to recognize GSCs would decrease upon their differentiation. Like NSCs, GSCs have the ability to differentiate into three downstream cell lineages: neuron, astrocyte and oligodendrocyte (9). However, differentiation efficiency and lineage choice vary significantly between each GSC line (15). To induce GSC differentiation, we first allowed tumorspheres to form during 6 days in presence of EGF and FGF-2, and then removed growth factors from culture medium and added 10% FBS. We noted that GSCs did not undergo cell death upon growth factor withdrawal, but instead tumorspheres attached to the plate and the cells started to migrate away from the sphere and change their morphology. To assess the efficacy of GSC differentiation, we analyzed stem *vs.* differentiation markers both at the levels of mRNA and protein expression after 4 days of differentiation. Of note, similar results were obtained using a 10-day differentiation protocol (data not shown). Following differentiation, GBM18 significantly downregulated *NES* (Nestin), a cytoskeletal protein typically expressed by NSCs and progenitor cells in developing brain (21), and *PROM1* (Prominin1, i.e., CD133), a cell surface marker of NCSs (22) (Figure 3A). In addition, we detected decreased expression of oligodendrocyte lineage markers (*OLIG2* and *PDGFRA*) in differentiated GBM18 cells, which was confirmed by flow cytometry (Figure 3A) and immunofluorescence analysis (Figure 3B). Compared with their stem-like counterparts, differentiated GBM18 cells showed an increase in CD24 levels, which has been correlated with neuronal differentiation and neuronal maturation (23) (Figure 3A). In GBM27 line, differentiation induced significant decrease of *NES* and *OLIG2* expression, paralleled by an increase in astrocyte markers *S100B* and *GFAP* (Figure 3C). Increased levels of GFAP after differentiation were confirmed by immunofluorescence analysis (Figure 3D), showing that GBM27 adopted an astrocyte phenotype. In addition, flow cytometry analysis revealed that differentiated GBM27 cells upregulated CD24 (Figure 3E), which can be found in neurons undergoing differentiation as well as astrocytes (24). In GBM38, differentiation induced an increase in CD24 levels, suggesting their neuronal differentiation (Figure 3F). Stem-like GBM38 cells showed relatively high levels of GFAP which was decreased after differentiation (Figure 3G). Conversely, the levels of OLIG2 increased, suggesting the presence of oligodendrocyte differentiation in GBM38 (Figure 3G). As evidenced by increased GFAP levels and decrease in *NES* and *OLIG2* expression, GBM123 differentiated into astrocytes (Figures 3H,I). Finally, GBM128B line evidently differentiated into neurons, as we detected decreased levels of *PDGFRA* and *OLIG2* together with increased expression of CD24 and *MAP2* (Figures 3J–L).

**FIGURE 4:**
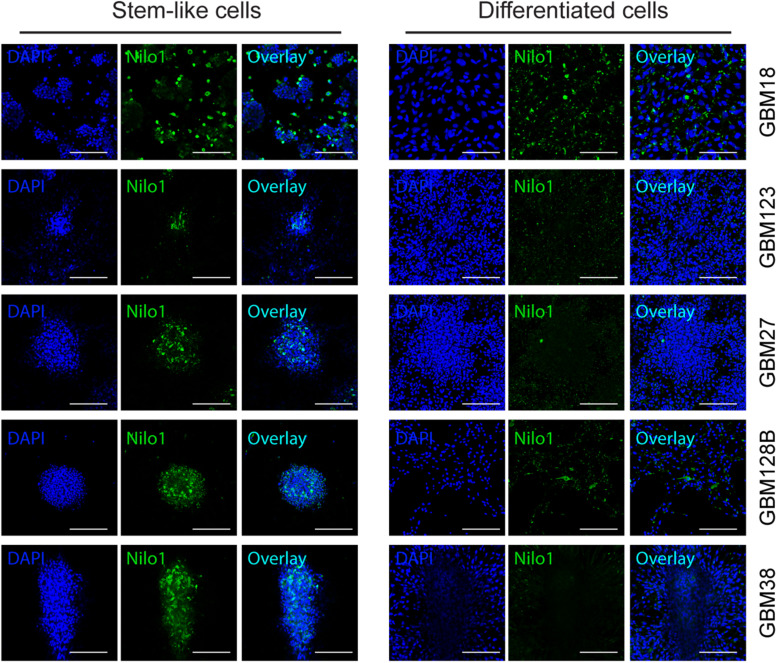
Nilo1 recognizes GSCs in stem-like state. GSCs were grown as in Figure 3. Confocal microscopy showing reduced proportions of Nilo-1 positive cells in differentiated GBM123, GBM27, GBM128B and GBM38 cells **(right)** compared with their stem-like equivalents **(left)**. Scale bar shows 100 μm.

We next analyzed the ability of Nilo1 to recognize GSCs cultured in stem cell conditions *vs.* those cultured in differentiation medium. Confocal microscopy (Figure 4) showed that, compared with tumorspheres grown in stem cell medium (left panels), the proportions of Nilo1-positive cells dramatically decreased along with the loss of stem cells upon GSC differentiation (right panels). Overall, these data showed that the decrease of Nilo1 ability to recognize GSCs correlates with the degree of their differentiation, which is in line with the hypothesis that Nilo1 identifies stem-like GSCs.

### Nilo1 Induces Apoptosis in GBM38

**FIGURE 5:**
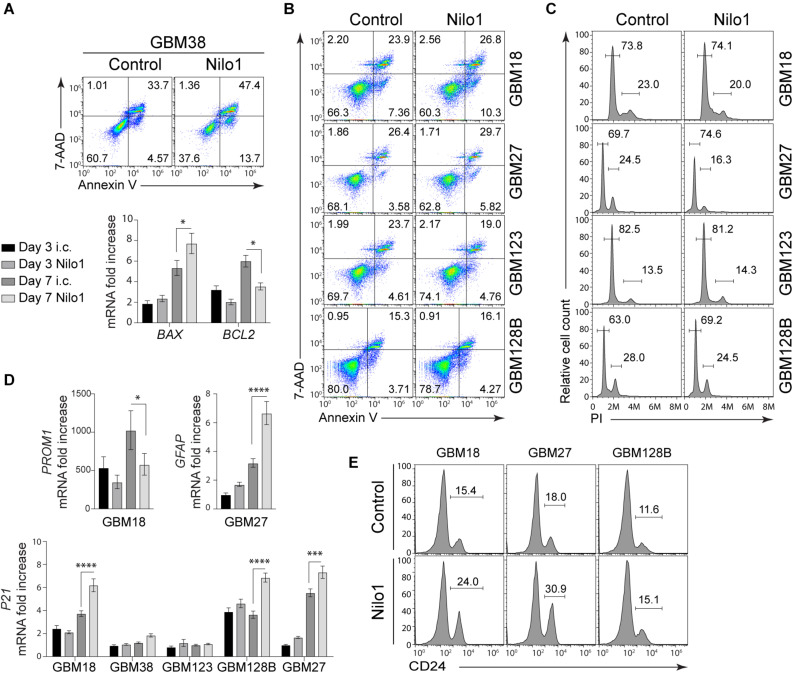
Nilo1 induces apoptosis and cell cycle arrest in GSCs. Neurospheres were dissociated and treated with Nilo1 (0.5 mg/ml) or with hamster anti-mouse CD3ε) as an irrelevant control (i.c.) for 7 days. **(A)** Flow cytometry analysis shows increased proportions of apoptotic (Annexin V^+^) cells and dead (PI^+^/Annexin V^+^) cells in GBM38 after Nilo1 treatment (upper panel). RT-PCR analysis shows increased *BAX* and decreased *BCL2* expression in GBM38, indicative of apoptosis (lower panel). **(B)** Flow cytometry analysis showing no significant change in apoptotic (Annexin V^+^) cells and dead (PI^+^/Annexin V^+^) cells in GBM18, GBM27, GBM123 and GBM128B after Nilo1 treatment. **(C)** Cell cycle analysis shows decreased proportions of proliferating (G2/M) and increased proportions of G1-arrested (G1/G0) GBM27 and GBM128B cells after Nilo1 treatment. **(D)** Nilo1 treatment reduces the expression of stemness marker *PROM1* in GBM18 and induces the expression of astrocyte marker *GFAP* in GBM27. The expression of *P21* is significantly induced in GBM18, GBM128B and GBM27 after 7-day Nilo1 treatment. **(E)** Flow cytometry analysis showing and increase of CD24, a neuronal differentiation marker, in GBM18, GBM27 and GBM128B after Nilo1 treatment. Panels **(A–C,E)** shown are representative histograms or dot plots of two experiments performed. Panels **(A)** and **(D)** data were normalized to GBM38 day 3 i.c. and show mean ± SD (*n* = 3), *p < 0.05, ****p < 0.0001, 1- or 2-way ANOVA (with Bonferroni correction).

The effect of Nilo1 treatment was the most evident in GBM38 cells, since it sharply reduced cell viability and completely abrogated sphere formation in these cells (Figures 1C, 2). To get an insight of the mechanism by which Nilo1 kills GBM38 cells, we tested the ability of Nilo1 to induce apoptosis. After 7 days of Nilo1 treatment, flow cytometry analysis revealed significantly increased AnnexinV^+^/7-AAD^+^ dead cell percentage (33 *vs.* 47%) in Nilo1-treated cells compared with controls (Figure 5A, upper panels). In addition, the percentage of apoptotic AnnexinV^+^/7-AAD^–^ cells was increased (4 vs. 13%), which suggested that Nilo1 acts through induction of apoptosis. To verify this, we assessed the ratio between apoptosis promoter *BAX* and apoptosis inhibitor *BCL2* expression, which is known to determine the cell’s susceptibility to die in response to apoptotic stimulus (25). Indeed, *BCL2* tended to decrease already at 3 days after Nilo1 treatment, and it was significantly decreased at 7 days, while the expression levels of *BAX* were significantly increased at this time point in Nilo1-treated cells compared with controls (Figure 5A, lower panels). These results demonstrated that Nilo1 inhibits GBM38 growth by inducing apoptosis.

### Nilo1 Arrests Cell Cycle in GBM18, GBM27, and GBM128B

We also tested the capacity of Nilo1 to induce cell death in other GSCs, however flow cytometry showed similar proportions of apoptotic and dead cells in GBM18, GBM27, GBM123 and GBM128B after 3 (data not shown) and 7 days of Nilo1 compared with control treatment (Figure 5B). As shown in Figure 1C, Nilo1 treatment reduced the numbers of viable cells in GBM27 and GBM128B, although these cells continued to grow during the course of the experiment. This finding indicated that Nilo1 might affect cell cycle progression in these cells, without having a pro-apoptotic effect. We therefore analyzed cell cycle profiles in GSCs, and found that Nilo1 treatment increased the percentage of cells in G0/G1 phase (69.7 vs. 74.6% in GBM27 and 63.0 vs. 69.2% in GBM128B, Figure 5C) compared to controls. In line with this, the proportions of proliferating cells in G2/M phase were reduced (24.5 vs. 16.3% in GBM27 and 28.0 vs. 24.5% in GBM128B, Figure 5C), suggesting that in presence of Nilo1 cells continue growing but at lower speed. This phenomenon of slowing down the cell cycle is known to occur during differentiation (26) and has been associated with neurogenic differentiation of neural stem-like progenitor cells during brain development (27). Therefore, these results, together with the fact that adherent cells were observed in Nilo1-treated GBM18 and GBM128B cells (Figure 2), led us to hypothesize that Nilo1 treatment might be inducing GSC differentiation. We thus examined the expression of stemness and differentiation markers, and found that Nilo1 treatment led to a reduction of Prominin1 (CD133) in GBM18, and induction of *GFAP* in GBM27 (Figure 5D). The induction of astrocyte-specific gene expression in GBM27 clearly pointed to a differentiation-inducing effect of Nilo1. Furthermore, RT-PCR analysis revealed that Nilo1 significantly induced the expression of p21 – a cyclin-dependent kinase 2 (CDK2) inhibitor – in GBM18, GBM27 and GBM128B (Figure 5D). Overexpression of cell cycle inhibitors is a key event ultimately leading to cell cycle arrest and induction of cell type-specific gene expression in cancer cells (28). In support of this, flow cytometry analysis showed that Nilo1 treatment significantly induced the expression of CD24, a marker of neuronal differentiation, in GBM18, GBM27, and GBM128B. Collectively, our findings thus point to a p21-dependent mechanism by which Nilo1 acts to reduce proliferation and induce differentiation in GSCs.

## Discussion

Nilo1 monoclonal antibody was generated against mouse neurospheres and it specifically recognizes NSCs in the mouse brain (13, 14). This antibody can be coupled with magnetic nanoparticles to identify mouse NSCs in their niche *in vivo*, and track their migration by MRI (magnetic resonance imaging) in response to brain damage (14). *In vitro*, Nilo1 treatment arrests mouse neurosphere proliferation, which suggested that Nilo1 targets a molecule functionally relevant for stem cell maintenance (13). In addition to mouse NSCs, Nilo1 was previously shown to recognize a homologous antigen within GBM patient-derived neurospheres (14). This raised the possibility that Nilo1 might be the first therapeutic drug targeting GSCs (29); however this remained to be investigated. In this work, we establish that, (1) Nilo1-specific targeting of GBM neurospheres depends on their stem-like phenotype, (2) Nilo1 affects the viability of GSCs but not normal stem cells, (3) in a portion of patient-derived GSCs Nilo1 treatment affects cell cycling and triggers differentiation in parallel with p21 induction, and (4) Nilo1 treatment kills a subset of patient-derived GSCs through a Bax-associated apoptotic mechanism.

Glioblastoma is characterized by extreme inter- and intra-tumoral heterogeneity, resulting in substantial differences in clinical characteristics and response to treatment. This heterogeneity is also reflected in GSC populations, showing different molecular and functional phenotypes that could explain why the effects of Nilo1 treatment varied between different GSC lines. For their growth and stemness maintenance, GSCs rely on a very complex network of signaling pathways, including Notch, Hedgehog and Wnt. Whether due to chromosomal instability, or different signaling events, yet our GSCs showed a remarkable difference in the expression of Notch, Hedgehog and Wnt downstream effectors (Supplementary Figure 5), which regulate key events in cell fate determination and proliferation. As there is considerable redundancy among these pathways, some GSCs lines might bypass the effects of Nilo1 and continue to grow despite blocking one route.

It would certainly be of relevance to find a marker defining Nilo1-sensitive GSC subtype. Currently, there is no universal marker for isolating GSCs or distinguishing them from NSCs. Our data showed that only GBM18 expressed CD133 (Prominin1), confirming that CD133 is not a universal marker for GSCs (30). GBM18, GBM27, and GBM123 expressed Nestin, while GBM128B showed the expression of PDGFRA and OLIG2. GBM38 lacked all putative stem cell markers except for GFAP, which is highly expressed in astrocytoma (31). Yet, GBM38 exhibits the most aggressive tumor growth *in vivo* (32) and was shown to be more resistant than other lines to a panel of drugs currently used in clinical practice (17). The fact that Nilo1 most effectively killed GBM38 cells therefore has twofold implications. First, our findings suggest that Nilo1 might be used in combination with current drugs to kill the most resistant GSCs. Second, in combination with therapeutic strategies that specifically target GSCS by their markers – such as CART (chimeric antigen receptor T cell) therapy – Nilo1 might aid to eliminate GSCs that lack most of the widespread CSC markers. We consider these possibilities worth investigating in future studies.

Glioblastoma stem-like cells, like normal stem cells, are resistant to conventional therapy that affects more differentiated cells of the bulk tumor. Therapies designed to specifically kill CSCs or target the pathways that maintain their stem-cell state and induce differentiation, might thus prove useful in the clinic. Because CSCs express specific markers, antibody-based therapies are considered as an effective approach to induce CSC cell death either directly, or to be used as antibody-drug conjugates. Indeed, anti-CD44 antibody can induce differentiation and apoptosis in leukemia and bladder cancer cells (33, 34), while CD133^+^ cancer cells can be targeted using an anti-CD133 antibody conjugated with cytotoxic drug (35). Nonetheless, there are currently no specific markers that tell apart CSCs from NSCs, and on-target/off-tumor toxicity represents one of the major challenges of CSC-targeted therapy. Nilo1 was first described as a monoclonal antibody that labels mouse NSCs (13), which raised the possibility that Nilo1 might also recognize NSCs in humans. This will certainly need to be explored in preclinical studies; nonetheless, our results showed that Nilo1 treatment did not have any toxic effects on normal human mesenchymal stem cells. To avoid its interaction with NSCs, using Nilo1 conjugated with gold nanoparticles was proposed for location-restricted photo-ablation therapy. In that case, any Nilo1 + NSCs would be protected by their location (in SVZ), which is distinct from the location of the tumor (36). Alternatively, recent studies revealed that glioblastomas may originate from NSCs of the SVZ that undergo malignant transformation and give rise to GSCs (12). Having this in mind, therapeutic strategies directed toward common molecular targets in NSCs and GSCs might be critical for achieving better prognosis for GBM patients.

An important factor for developing antibody-based therapies is the presence of blood–brain barrier (BBB). Intact BBB efficiently prevents crossing of systemically administered drugs and monoclonal antibodies larger than 400 Da from the bloodstream into the brain parenchyma (37). GBM is often characterized by a disruption of BBB and open endothelial tight junctions, which allow limited amounts of drugs to reach the tumor site. Nonetheless, the extent of BBB disruption is difficult to determine and varies from patient to patient. To bypass BBB, locoregional delivery using intracerebral placement of catheters can be used as a strategy for enhancing intraparenchymal drug delivery (37). Pharmacodynamics and pharmacokinetics studies will need to be performed to determine the *in vivo* efficacy of Nilo1 antibody.

Our results establish Nilo1 as a potential therapeutic drug targeting GSCs, with several possible applications. First, our data show that Nilo1 labels GSCs, hence conjugating Nilo1 with a dye might allow for better visualization of highly infiltrative GBM tumors. Second, Nilo1 treatment directly induced cell death in one patient-derived GSC line. In addition, the fact that Nilo1 treatment disabled sphere formation and triggered differentiation in a subset of patient-derived GSCs, suggested that these cells, by losing self-renewal and stemness properties, might become susceptible to standard chemotherapy and radiotherapy. Finally, the fact that all tested GSC lines were Nilo1-positive raises the possibility that conjugating Nilo1 with a cytotoxic drug might encompass the full spectrum of GSC molecular subtypes. We believe that future studies exploring this wide range of possible applications might set the stage for Nilo1 humanized antibody development and clinical trials.

## Data Availability Statement

The datasets presented in this study can be found in online repositories. The names of the repository/repositories and accession number(s) can be found in the article/ Supplementary Material.

## Ethics Statement

All patients gave informed consent and the use of tumor samples was approved by the Hospital La Fe (Spain) Ethics Committee.

## Author Contributions

ÁA-S and VG-R conceived the study. ÁA-S, VG-R, and GR designed most of the experiments, analyzed the data, and wrote the manuscript. GR performed most of the experiments, with the help of GI, DU, PP, and CQ. CE-L provided the mesenchymal stem cells. All authors read, discussed, and approved the manuscript.

## Conflict of Interest

AS was employed by the MSD Company, now retired. PP and VG-R were employed by the Atrys Health. The remaining authors declare that the research was conducted in the absence of any commercial or financial relationships that could be construed as a potential conflict of interest. The reviewer CB declared a shared affiliation, with no collaboration, with one of the authors CE-L to the handling editor at the time of review.

## Supplementary Material

FIGURE S1 Cell cycle analysis for GBM38 line at different time points during neurosphere formation.

FIGURE S2 MTS assay showing that Nilo1 treatment does not affect the viability of mesenchymal stem cells. Data were normalized to day 0 and show mean ± SD (*n* = 3).

FIGURE S3 Sphere formation assay at day 14, showing that Nilo1 treatment impedes sphere formation in GBM18, GBM128B, and GBM38. Scale bar shows 100 μm.

FIGURE S4 Immunofluorescence analysis of GBM27, GBM38 and GBM128B tumorspheres showing the proportions of non-viable cells stained with 7-AAD. Scale bar shows 50 μm.

FIGURE S5 RT-PCR showing different expression levels of Hedgehog (*GLI1*), Notch (*HEY2*), and Wnt (*BMP4*) downstream effectors in GSC lines.
