# Supplementary figures and images for: Potential Therapeutic Effects of the Neural Stem Cell-Targeting Antibody Nilo1 in Patient-Derived Glioblastoma Stem Cells
Source: Front Oncol. 2020 Aug 14;10:1665. doi: 10.3389/fonc.2020.01665 (PMC7468525; doi:10.3389/fonc.2020.01665)

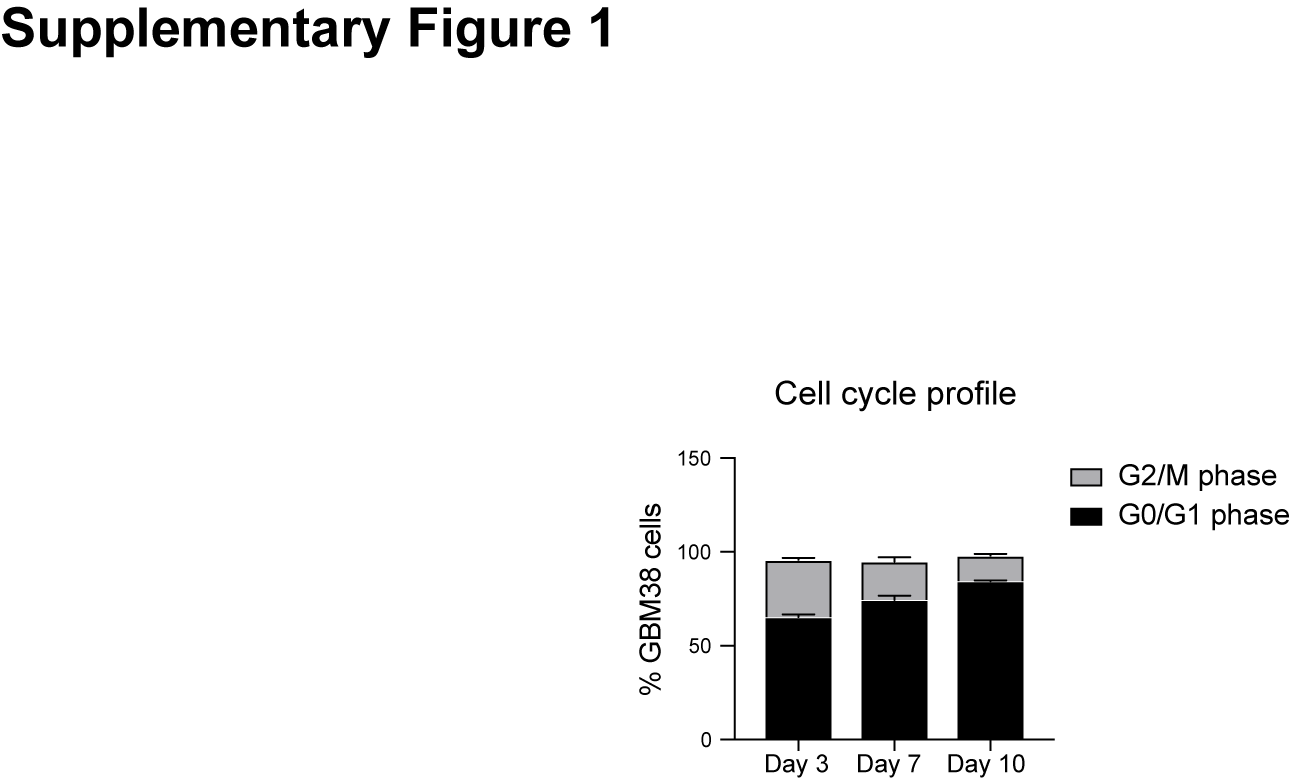

Supplement: FIGURE S1 — Cell cycle analysis for GBM38 line at different time points during neurosphere formation. [file Image_1.TIF]

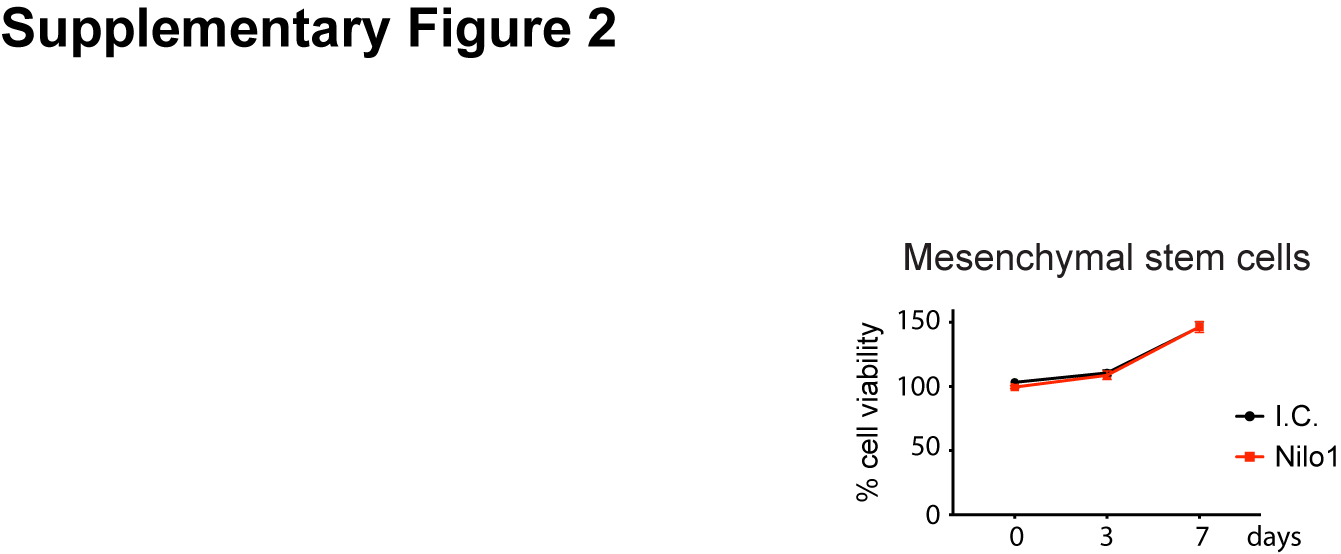

Supplement: FIGURE S2 — MTS assay showing that Nilo1 treatment does not affect the viability of mesenchymal stem cells. Data were normalized to day 0 and show mean ± SD (n = 3). [file Image_2.TIF]

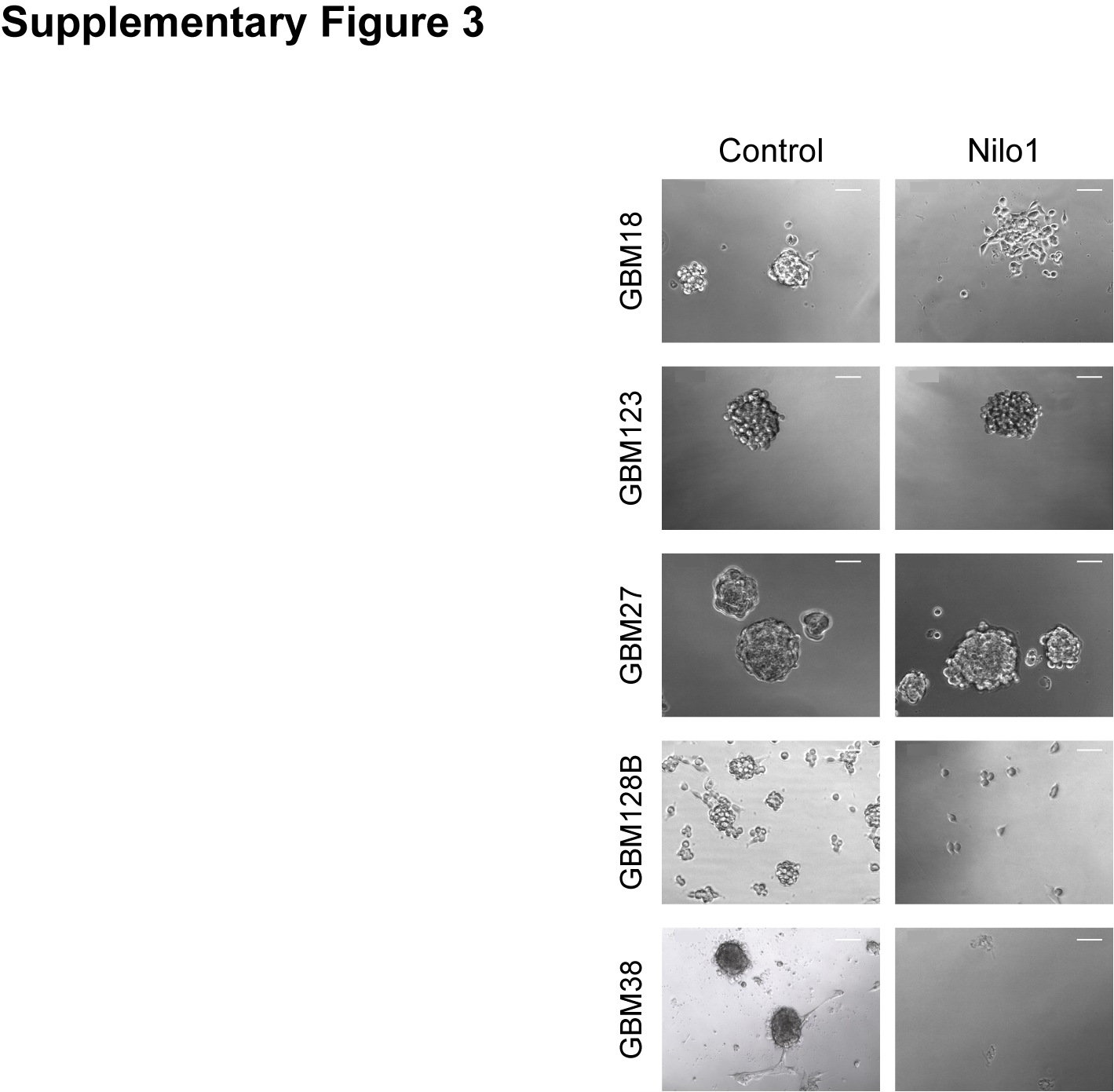

Supplement: FIGURE S3 — Sphere formation assay at day 14, showing that Nilo1 treatment impedes sphere formation in GBM18, GBM128B, and GBM38. Scale bar shows 100 μm. [file Image_3.TIF]

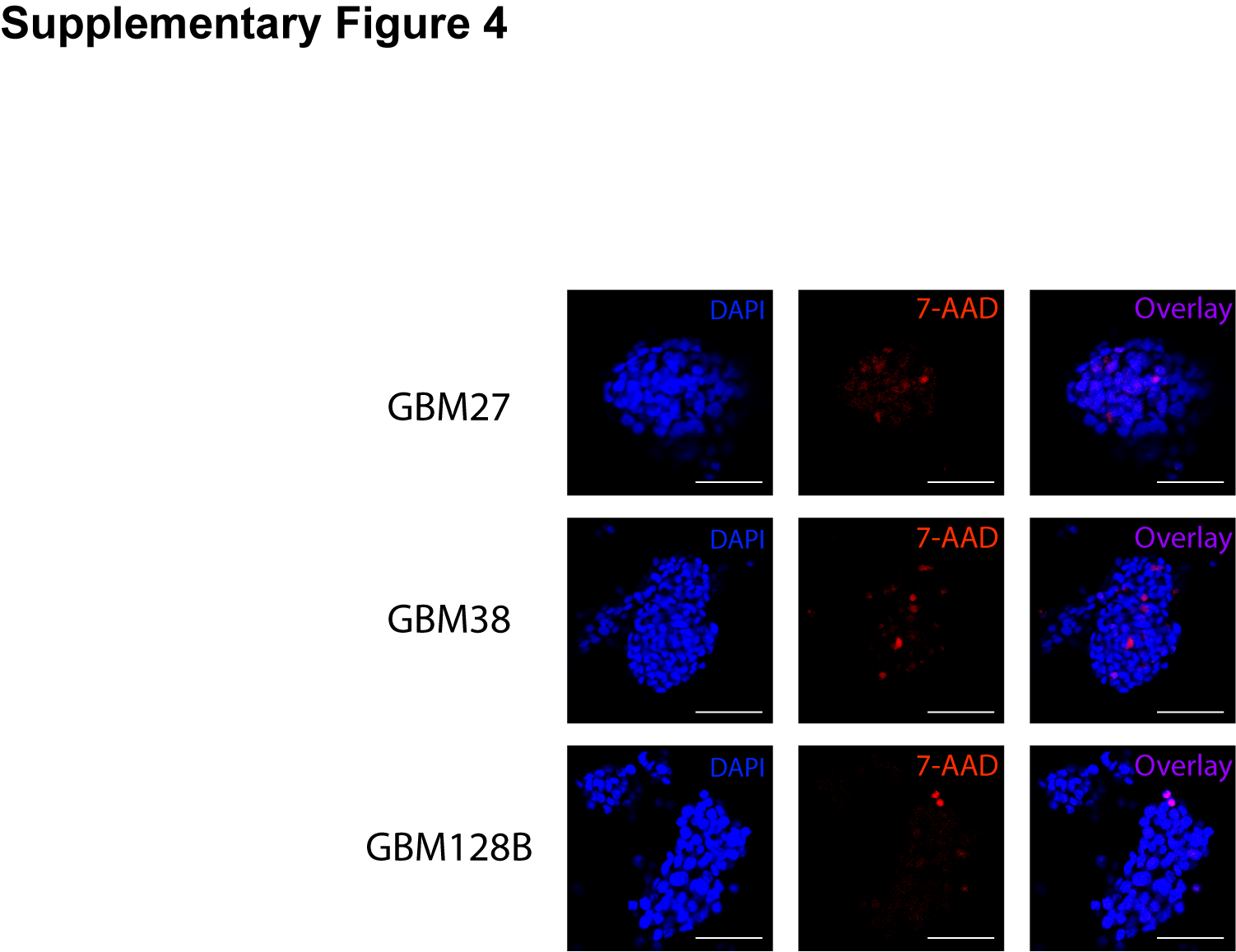

Supplement: FIGURE S4 — Immunofluorescence analysis of GBM27, GBM38 and GBM128B tumorspheres showing the proportions of non-viable cells stained with 7-AAD. Scale bar shows 50 μm. [file Image_4.TIF]

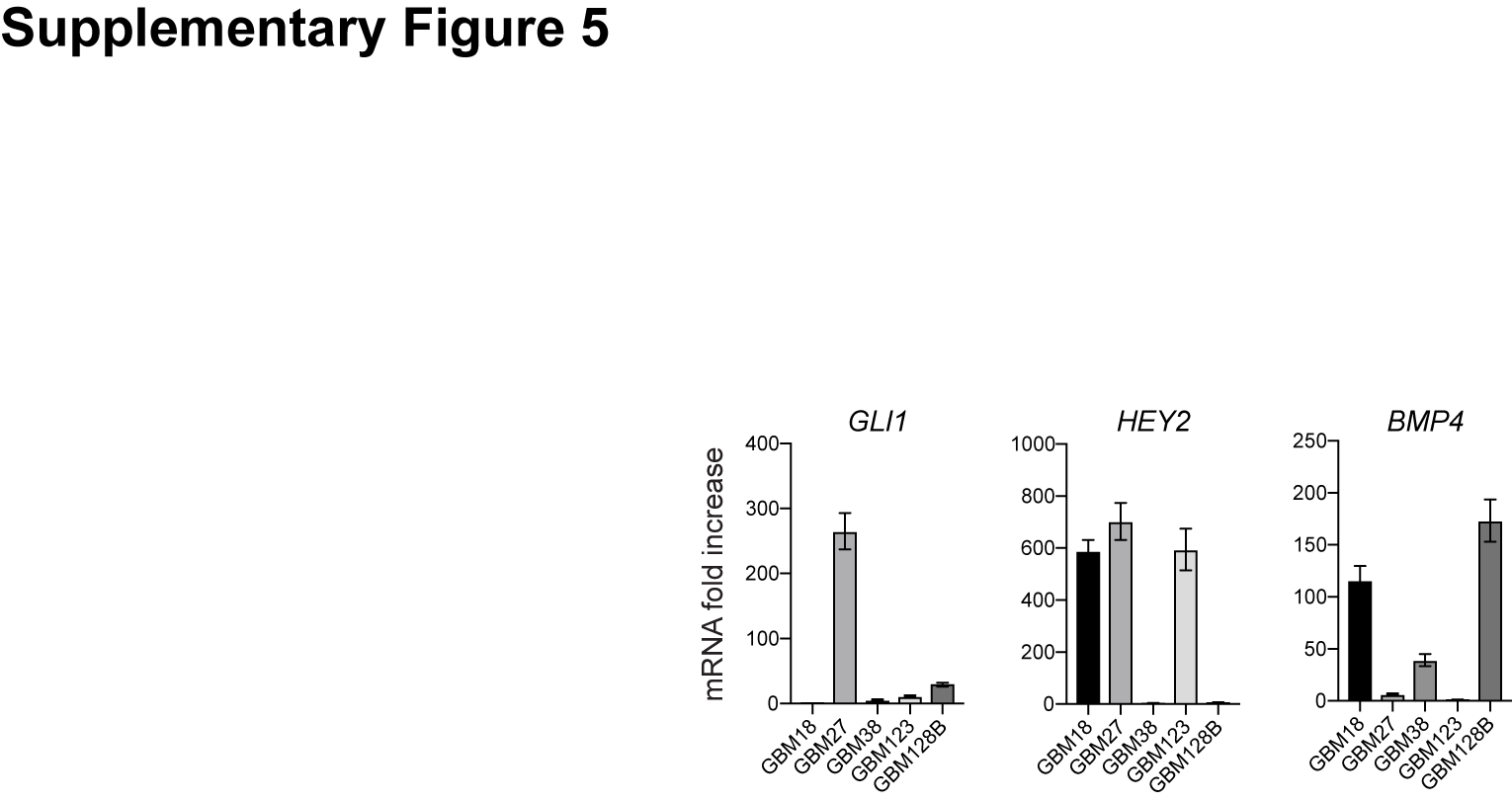

Supplement: FIGURE S5 — RT-PCR showing different expression levels of Hedgehog (GLI1), Notch (HEY2), and Wnt (BMP4) downstream effectors in GSC lines. [file Image_5.TIF]
